# Risk factors and prognosis analysis of gastrointestinal bleeding combined with hemorrhagic shock in patients in intensive care unit:A retrospective study

**DOI:** 10.1371/journal.pone.0348276

**Published:** 2026-05-05

**Authors:** Changfu Liu, Jinju Li

**Affiliations:** Department of Intensive Care Medicine, Bengbu First People’s Hospital, Anhui Province, China; Azienda Ospedaliero Universitaria Careggi, ITALY

## Abstract

**Background:**

Through retrospective analysis of clinical data on gastrointestinal bleeding complicated with hemorrhagic shock (GIB-HS) in patients in our intensive care unit（ICU）, we aim to explore relevant risk factors and prognosis, and provide guidance for clinical prevention and treatment of such cases.

**Methods:**

A total of 93 cases of gastrointestinal bleeding from October 2023 to January 2025 in the Intensive Care Medicine Department of our hospital were collected. Based on the presence or absence of hemorrhagic shock, they were divided into a hemorrhagic shock group (observation group, n = 42 cases) and a control group (control group, n = 51 cases). The differences in clinical data between the two groups were analyzed, and logistic regression was used to analyze the risk factors for gastrointestinal bleeding complicated with hemorrhagic shock. The sensitivity and specificity of predicting hemorrhagic shock were analyzed using the receiver operating characteristic（ROC）curve, and the prognosis of the two groups was compared.

**Results:**

There were statistically significant differences in the scores of type 2 diabetes, urea nitrogen, red blood cell number, hemoglobin, hematocrit and Acute Physiology and Chronic Health Evaluation II(APACHEII) score, *P* ＜ 0.05. Binary logistic regression analysis was conducted with factors with *P* < 0.1 as independent variables. The results showed that there was a statistically significant difference in urea nitrogen and fibrinogen, which were risk factors for ICU GIB-HS The ROC curve showed that urea nitrogen had the highest specificity in predicting ICU patients with GIB-HS, while fibrinogen had the highest sensitivity in predicting hemorrhagic shock. Compared with the prognosis of the two groups, the mortality rate of the group with hemorrhagic shock was higher than that of the group without hemorrhagic shock.

**Conclusions:**

An exploratory correlation analysis revealed that both urea nitrogen and fibrinogen are factors associated with the prediction of GIB-HS in ICU patients, while APACHEII score, respiratory failure, and fibrinogen are independent factors affecting the prognosis of ICU patients with GIB-HS. Clinicians need to timely identify these indicators and optimize treatment plans to improve prognosis.

1. Patients with advanced malignant tumors, 2. Age of onset<18 years, 3. Pregnant or lactating women, 4. Incomplete case or follow-up data.

## 1. Introduction

In the intensive care unit, critically ill patients often have gastrointestinal bleeding, which can be divided into upper gastrointestinal bleeding and lower gastrointestinal bleeding according to the location of occurrence. The causes of upper gastrointestinal bleeding mainly include ulcers, gastroesophageal varices, and cancer, while the causes of lower gastrointestinal bleeding include ulcerative colitis, enteritis, hemorrhoids, diverticular bleeding, rectal ulcers, and colorectal cancer [1]. Research has shown that in the emergency room, using the Glasgow Brachford score can stratify the risk of gastrointestinal bleeding and is superior to the Rockall score in predicting clinical outcomes for upper gastrointestinal bleeding patients in the United States [2]. Some researchers have used the shock index, which is the ratio of heart rate to systolic blood pressure, as a valuable tool for predicting the mortality rate of gastrointestinal bleeding [3]. When the amount of gastrointestinal bleeding in patients is small, fasting and medication that inhibits gastric acid secretion can often stop the bleeding. However, when the amount of gastrointestinal bleeding is large or rapid, patients often experience a decrease in blood pressure or even shock, and this condition is life-threatening. At present, there is no clear definition of gastrointestinal bleeding. Some researchers define gastrointestinal bleeding as any bleeding that causes hemodynamic instability, signs of poor tissue perfusion (such as changes in mental state, fainting, or pale complexion), transfusion of more than 2 units of red blood cells during initial fluid resuscitation, or significant and rapid gastrointestinal bleeding. Although the mortality rate of patients with gastrointestinal bleeding is between 3% and 14%, the mortality rate of gastrointestinal bleeding is much higher, and the mortality rate is related to liver cirrhosis, severe complications such as chronic kidney disease, and alcohol consumption [4]. At present, proton pump inhibitors, erythromycin, and red blood cell transfusions are given based on risk stratification in treatment. Endoscopic treatment is selectively performed according to the indications for endoscopic examination, and tranexamic acid should not be used [5]. In the ICU, end-stage heart failure patients are commonly treated with left ventricular assist devices, which often result in gastrointestinal bleeding with an incidence rate of 15% −25%. For these patients, treatment measures include maintaining hemodynamic stability, maintaining or reversing antithrombotic therapy, or stopping bleeding through endoscopy and interventional methods. Prophylactic use of octreotide and danazol can reduce gastrointestinal bleeding caused by left ventricular assist devices by reducing arteriovenous malformations [6]. Given that gastrointestinal bleeding is a common and frequently occurring disease, and the condition rapidly deteriorates after the onset of hemorrhagic shock, this study focuses on analyzing the risk factors for GIB-HS in patients in intensive care units, as well as the prognostic factors affecting hemorrhagic shock patients and the sensitivity and specificity indicators for predicting the prognosis of such patients.

## 2. Materials and methods

### 2.1. Study population

This study is a retrospective study, selecting 93 cases of gastrointestinal bleeding in the Intensive Care Medicine Department of the First People#39;s Hospital of Bengbu City. The time range for collecting cases is between October 2023 and January 2025, and the author is authorized to access the data of study individuals during and after the study period, but identifiable identity information is anonymized. The research project was approved by the Ethics Committee of the First People#39;s Hospital of Bengbu City (Ethical Number：BBYYKTPJ2025018).The research subjects of this project do not include minors. All study subjects (signed by family representatives if the patient does not have autonomous behavioral ability) have signed a written informed consent form agreeing to use their medical data for the study. This study has been conducted according to the principles expressed in the Declaration of Helsinki.

### 2.2. Inclusion criteria

1. Meet the diagnosis of gastrointestinal bleeding, 2. Age of onset ≥ 18 years, 3. Complete case data.

### 2.3. Exclusion criteria

**Fig 1:**
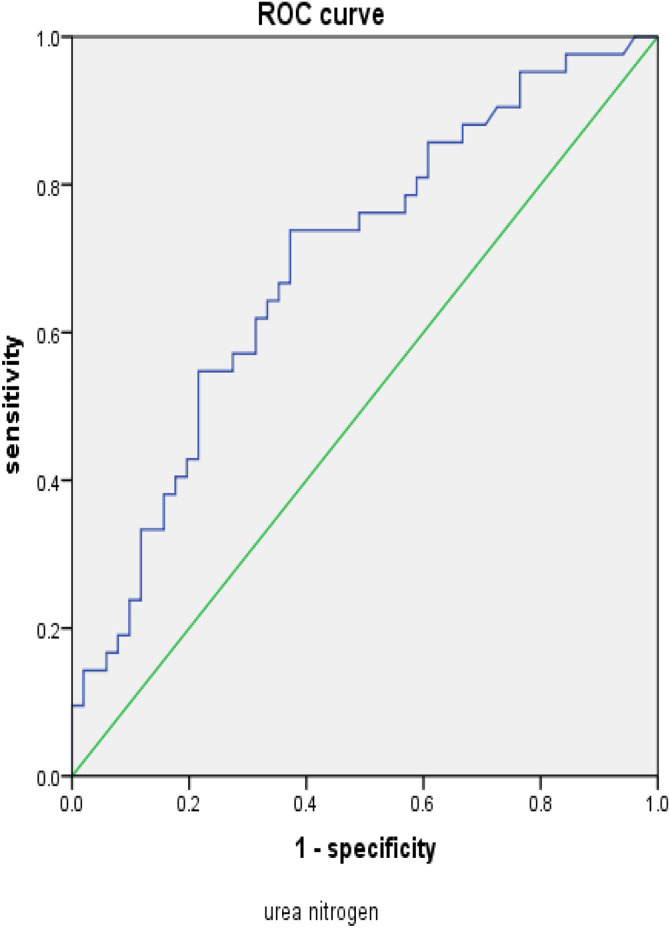
ROC curve related to urea nitrogen.

### 2.4. Grouping method

According to whether hemorrhagic shock occurred, they were divided into a hemorrhagic shock group (observation group, n = 42 cases) and a non hemorrhagic shock group (control group, n = 51 cases). Hemorrhagic shock is a critical syndrome caused by acute massive blood loss, leading to a sharp reduction in effective circulating blood volume, resulting in inadequate tissue perfusion, cellular metabolic dysfunction, and organ impairment. Its core diagnostic criteria typically include circulatory and volume parameters: systolic blood pressure below 90 mmHg or a drop of more than 40 mmHg from baseline; central venous pressure (CVP) often below 5 mmHg; and tachycardia (heart rate > 100 beats/min).

### 2.5. Data collection and clinical variables

The data used in this study were accessed on 02/06/2025 (June 2, 2025). Collect the basic information of patients, including: gender, age, APACHEII score, basic diseases (hypertension, diabetes, etc.), respiratory failure, hospitalization expenses, self-care ability score, days of hospitalization, whether there is shock, number of white blood cells, number of neutrophils, lymphocyte count, monocyte count, number of red blood cells, hemoglobin, hematocrit, urea nitrogen, creatinine, fibrinogen, troponin I, D-dimer, and record the prognosis of patients (the 30-day mortality rate).

### 2.6. Conventional treatment

Including fasting, intravenous application of proton pump inhibitors, use of somatostatin or octreotide, fluid resuscitation, blood transfusion, vasoactive drugs, and other treatments.

### 2.7. Follow up time

One month after discharge, the patient#39;s current condition will be assessed through telephone follow-up by trained follow-up personnel.

### 2.8. Statistical methods

SPSS21 statistical software was used for analysis. All quantitative data in this study were expressed in the form of mean ± standard deviation. Independent sample t-test was used to compare the two groups of data; All count data are expressed in terms of quantity and percentage in the study population, using a cross tabulation chi square test. Perform logistic regression analysis on the single factors with statistically significant differences to calculate their OR values and 95% CI. *P* < 0.05 indicates a statistically significant difference. The statistical software used in this study is SPSS21.0. Draw the work characteristic curve of the subjects to analyze the sensitivity and specificity of relevant risk factors.

## 3. Results

### 3.1. Univariate comparative analysis of clinical data between two groups (Table 1,2)

There were no significant differences in sex, age, hypertension, respiratory failure, hospitalization costs, self-care ability score, length of hospital stay, white blood cell count, neutrophil count, lymphocyte count, monocyte count, creatinine, fibrinogen, troponin I, and D-dimer between the two groups, P ＞ 0.05； There were statistically significant differences in diabetes, urea nitrogen, number of red blood cells, hemoglobin, hematocrit and APACHEII scores, *P* ＜ 0.05.

**Table 1:** Independent sample t-test results of continuous variable univariate analysis of gastrointestinal bleeding (with or without hemorrhagic shock) in ICU patients.

factor	T value	*P* value
Hospitalization expenses	0.884	0.379
Life self-care ability score	0.951	0.345
age	−1.355	0.179
Hospitalization days	0.402	0.689
APACHEII score	2.464	0.016
white blood cell count	−0.010	0.992
Neutrophil count	0.417	0.678
Mononuclear cell count	−0.695	0.489
Lymphocyte count	−1.214	0.228
red blood cell count	−2.395	0.019
hemoglobin	−2.747	0.007
hematocrit	−2.812	0.006
Platelet count	−1.133	0.261
urea nitrogen	2.895	0.005
creatinine	1.775	0.079
Troponin I	−0.867	0.388
fibrinogen	−1.847	0.068
D-Dimer	0.937	0.351

Independent sample t-test shows statistically significant differences among urea nitrogen, number of red blood cells, hemoglobin, hematocrit and APACHEII score(*P* ＜ 0.05)

**Table 2:** Cross tabulation chi square test for count data of gastrointestinal bleeding (with or without hemorrhagic shock) in ICU patients.

factor		Hemorrhagic shock group	Non hemorrhagic shock group	Chi square test F-value	*P* value
hypertension	yes	23	21	1.705	*P* = 0.192
	no	19	30		
type 2 diabetes	yes	12	37	17.870	*P* = 0.000
	no	30	14		
respiratory failure	yes	30	36	0.008	*P* = 0.929
	no	12	15		
sex	male	28	34	0	*P* = 1.0
	female	14	17		
prognosis	alive	17	32	4.582	*P* = 0.032
	dead	25	19		

Cross table chi square test showed that type 2 diabetes was statistically significant(*P* ＜ 0.05).

### 3.2 Analysis of risk factors of GIB-HS (Table 3)

Binary logistic regression analysis was conducted for all the above single factors with P < 0.1, and the dependent variable was hemorrhagic shock (1 for yes, 0 for no), and the independent variable was: type 2 diabetes, creatinine, urinary nitrogen, APACHEII score, fibrinogen, number of red blood cells, hemoglobin, hematocrit. The results showed that the difference between urea nitrogen and fibrinogen was statistically significant, P ＜ 0.05， Risk factors for ICU GIB-HS.

**Table 3:** Binary logistic regression analysis of hemorrhagic shock caused by ICU gastrointestinal bleeding.

factor	B	S.E	Wals	*P*	Exp(B)	95% CI lower limit	95% CI upper limit
urea nitrogen	0.072	0.025	8.378	0.004	1.074	1.023	1.128
fibrinogen	−0.228	0.106	4.606	0.032	0.796	0.646	0.980

Binary logistic regression analysis shows statistically significant differences in urea nitrogen and fibrinogen levels(*P* ＜ 0.05).

### 3.3. Prognostic analysis of two groups

17 cases improved and 25 cases died in the hemorrhagic shock group, while 32 cases improved and 19 cases died in the group without hemorrhagic shock. The mortality rate in the hemorrhagic shock group was significantly higher than that in the group without hemorrhagic shock. Cross tabulation chi square test was performed between the two groups, *P* = 0.032， The difference is statistically significant.

### 3.4. Sensitivity and specificity of predicting ICU GIB-HS (Fig 1,2)

The sensitivity and specificity of predicting hemorrhagic shock in ICU patients with gastrointestinal bleeding are as follows: the area under the urea nitrogen curve is 0.693, *P* = 0.001, The optimal value is 9.195, with a sensitivity of 73.8%, specificity of 62.7%, and an area under the fibrinogen scoring curve of 0.621, *P* = 0.045， The optimal value is 4.49, with a sensitivity of 78.6% and a specificity of 52.9%. It can be concluded that both urea nitrogen and fibrinogen can effectively predict the risk factors for gastrointestinal bleeding and hemorrhagic shock in ICU patients.

**Fig 2:**
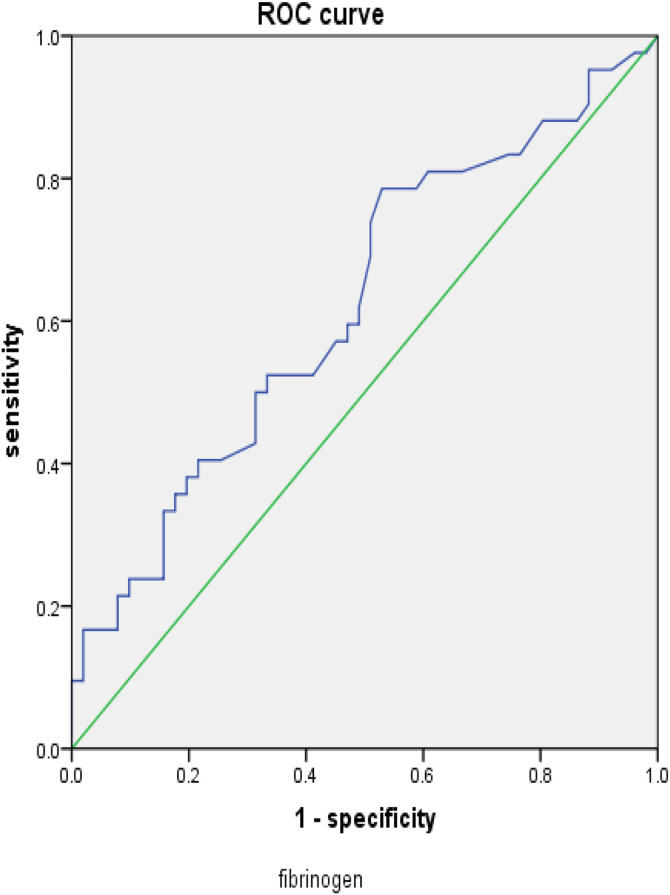
ROC curve related to fibrinogen.

### 3.5. Analysis of prognostic factors in the group of gastrointestinal bleeding complicated with hemorrhagic shock

Through univariate analysis of each factor (Table 4,5), it was found that respiratory failure, APACHEII score, and fibrinogen were prognostic factors with *P* < 0.05. The *P* value of D-dimer was 0.082, and the *P* value of platelet count was 0.09. The above five factors *(P* < 0.1) were set as independent variables for binary logistic regression analysis, and the prognosis was set as the dependent variable. It was found that APACHEII score, respiratory failure, and fibrinogen were statistically significant, *P* ＜ 0.05，The independent influencing factors for the prognosis of ICU patients with gastrointestinal bleeding complicated by hemorrhagic shock are shown in Table 6.

**Table 4:** Univariate analysis of categorical variables in ICU gastrointestinal bleeding complicated with hemorrhagic shock group.

factor		Prognosis: alive(case)	Prognosis: dead(case)	Cross tabulation chi square test(F value)	*P* value
hypertension	yes	15	8	0.684	*P* = 0.408
	no	10	9		
type 2 diabetes	yes	6	6	0.2	*P* = 0.655
	no	19	11		
respiratory failure	yes	23	7	10.438	*P* = 0.001
	no	2	10		

Cross table chi square test showed that respiratory failure was statistically significant(*P* ＜ 0.05)

**Table 5:** Independent sample t-test results of continuous variable factors in ICU gastrointestinal bleeding complicated with hemorrhagic shock group.

factor	T value	*P* value
Hospitalization expenses	0.466	0.644
age	0.975	0.336
Hospitalization days	1.172	0.248
APACHEII score	−4.442	0.000
white blood cell count	−0.693	0.494
Neutrophil count	−0.994	0.326
Mononuclear cell count	0.455	0.652
Lymphocyte count	0.789	0.435
red blood cell count	−1.339	0.188
hemoglobin	−0.749	0.459
hematocrit	−0.904	0.372
Platelet count	1.738	0.09
urea nitrogen	0.758	0.453
creatinine	−0.300	0.766
Troponin I	−1.109	0.274
fibrinogen	2.754	0.009
D-Dimer	−1.817	0.082

Independent sample t-test showed that APACHEII score, and fibrinogen were prognostic factors with *P* < 0.05.

**Table 6:** Binary logistic regression analysis of ICU gastrointestinal bleeding complicated with hemorrhagic shock group.

factor	B	S.E	Wals	*P*	Exp(B)	95% CI lower limit	95% CI upper limit
APACHEII score	−0.131	0.046	7.975	0.005	0.877	0.801	0.961
respiratory failure	2.472	0.944	6.852	0.009	11.849	1.861	75.447
fibrinogen	0.512	0.225	5.164	0.023	1.668	1.073	2.594

Binary logistic regression analysis showed that APACHEII score, respiratory failure, and fibrinogen were statistically significant, *P* ＜ 0.05.

### 3.6. Sensitivity and specificity of predicting the prognosis of GIB-HS in ICU patients (Fig 3,4)

The sensitivity and specificity of predicting the prognosis of GIB-HS in ICU patients by displaying the APACHEII score, respiratory failure, and fibrinogen risk factors in the subject#39;s working characteristic curve are as follows: the area under the APACHEII score curve is 0.832, *P* = 0.000, The optimal value is 25.5, with a sensitivity of 60%, specificity of 94.1%, and an area under the fibrinogen scoring curve of 0.714, *P* = 0.020， The optimal value is 4.17, with a sensitivity of 52.9% and a specificity of 84%. Therefore, it can be concluded that both APACHEII score and fibrinogen can effectively predict the prognosis of ICU gastrointestinal bleeding patients with hemorrhagic shock.

**Fig 3:**
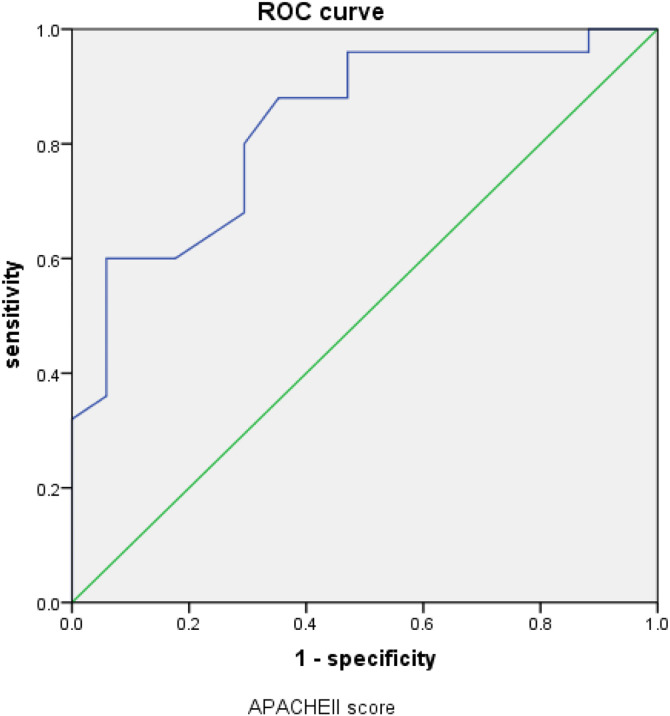
ROC curve related toAPACHEII score.

**Fig 4:**
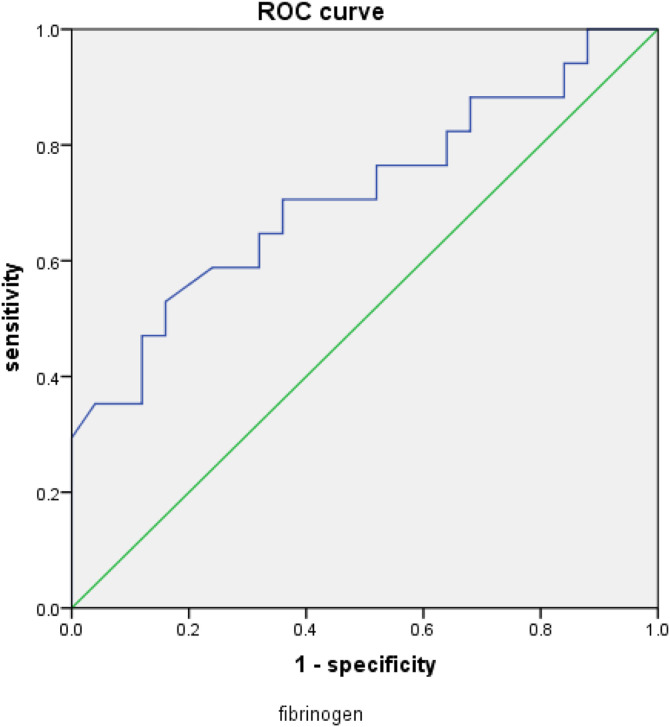
ROC curve related to fibrinogen.

## 4. Discussion

When gastrointestinal bleeding occurs rapidly and in large amounts, patients will experience a decrease in blood pressure and even shock. Studies have shown that the overall proportion of patients with gastrointestinal bleeding experiencing shock or hemodynamic instability is 25%. In variceal bleeding, this proportion is 25%, while in non variceal bleeding, this proportion is 22%. The proportion of patients with colonic diverticulum bleeding experiencing shock or hemodynamic instability is 12% [7]. Urea nitrogen, as a protein metabolite, is commonly elevated in cases of urea excretion disorders and excessive urea production. Urea excretion disorders are common in renal dysfunction and urinary tract obstructive diseases, while excessive urea production is common in high protein diets, tissue necrosis, and gastrointestinal bleeding. During gastrointestinal bleeding, the lost protein in the blood and necrotic tissue at the bleeding site are absorbed by the intestine, and the body is in a stress state during bleeding. Protein breakdown metabolism is enhanced, effective circulating blood volume is insufficient, renal perfusion is reduced, and urine output is reduced, all of which can lead to an increase in blood urea nitrogen. Studies have shown that the ratio of urea nitrogen to creatinine can serve as a diagnostic indicator for whether gastrointestinal bleeding requires intervention [8]. This study found through binary logistic regression that urea nitrogen is an independent influencing factor for the occurrence of hemorrhagic shock in gastrointestinal bleeding. The ROC curve showed that urea nitrogen can be used as an observation indicator to predict the occurrence of hemorrhagic shock in gastrointestinal bleeding, with an area under the ROC curve of 0.693, *P* = 0.001, The optimal value is 9.195, with a sensitivity of 73.8% and specificity of 62.7%. The urea nitrogen value is positively correlated with the risk of conversion to hemorrhagic shock, and the higher the urea nitrogen value, the higher the risk of conversion to hemorrhagic shock.

The APACHEII score, as a scoring system, is an indicator for assessing the severity of severe illness in critically ill patients and predicting prognosis. It is widely used in clinical practice [9]. It consists of three parts: age score, acute physiology score, and chronic health score, with a maximum score of 71 points. Generally, the higher the score, the more severe the condition [10]. This study included the APACHEII scoring index in the prognostic analysis of the group with GIB-HS. Through binary logistic regression analysis, it was found that the APACHEII score was an independent influencing factor on the prognosis of the group with gastrointestinal bleeding complicated by hemorrhagic shock. The ROC curve showed that the APACHEII score could be used as an observation index to predict the group with gastrointestinal bleeding complicated by hemorrhagic shock. The area under the ROC curve was 0.832, *P* = 0.000, The optimal value is 25.5, with a sensitivity of 60% and a specificity of 94.1%. The APACHEII score is negatively correlated with the prognosis of the hemorrhagic shock group. The higher the APACHEII score is positively associated with mortality. Patients with APACHEII scores below 25.5 have better prognosis than those with scores above 25.5.

Fibrinogen is a glycoprotein synthesized and secreted by liver cells with coagulation function. Under the action of thrombin, fibrinogen is converted into fibrin and directly participates in the coagulation process. When gastrointestinal bleeding occurs, fibrinogen is directly consumed. If accompanied by cirrhosis or hepatitis, the synthesis of fibrinogen is reduced, which can lead to a decrease in fibrinogen. Studies have shown that low fibrinogen levels and platelet aggregation dysfunction may predict the risk of gastrointestinal bleeding in children with decompensated cirrhosis [11]. This study found through binary logistic regression that fibrinogen is an independent influencing factor of hemorrhagic shock in gastrointestinal bleeding. The ROC curve showed that fibrinogen can be used as an observation indicator to predict the occurrence of hemorrhagic shock in gastrointestinal bleeding. The area under the ROC curve was 0.621, *P* = 0.045，The optimal value is 4.49, with a sensitivity of 78.6% and specificity of 52.9%. Fibrinogen is negatively correlated with the risk of conversion to hemorrhagic shock, with lower fibrinogen levels indicating a higher risk of conversion to hemorrhagic shock; When analyzing the prognosis of the group with gastrointestinal bleeding and hemorrhagic shock, binary logistic regression analysis revealed that fibrinogen was an independent factor affecting the prognosis of the group with gastrointestinal bleeding and hemorrhagic shock. The ROC curve showed that fibrinogen score could be used as an observation indicator for predicting the prognosis of the group with gastrointestinal bleeding and hemorrhagic shock. The area under the ROC curve was 0.714, *P* = 0.020， The optimal value is 4.17, with a sensitivity of 52.9% and a specificity of 84%.

In this study, we have included respiratory failure indicators into the observation indicators of gastrointestinal hemorrhage with hemorrhagic shock. Respiratory failure refers to the serious impairment of pulmonary ventilation and (or) pulmonary ventilation function caused by various diseases, leading to hypoxia with (or without) carbon dioxide retention. In the intensive care unit, many diseases will eventually develop respiratory failure. Blood gas analysis shows that the blood oxygen partial pressure is low, with or without elevated carbon dioxide partial pressure. At this time, the body is in a stress state, and the sympathetic adrenal medullary system is activated, leading to increased catecholamine production, causing peripheral and visceral vasoconstriction, gastrointestinal vasoconstriction is obvious, leading to ischemia and hypoxia of gastrointestinal mucosa, mucosal barrier function damage, erosion, ulcer, and digestion Road bleeding, This study included respiratory failure indicators in the prognostic analysis of GIB-HS group. Through binary logistic regression analysis, it was found that respiratory failure was an independent factor affecting the prognosis of GIB-HS group.

Limitations of this study: This study is a single center retrospective study with a small number of cases. Compared to the Glasgow Brachford score and Rockal score for predicting the prognosis of gastrointestinal bleeding, this study selected fewer influencing factors. The risk factors for concurrent hemorrhagic shock need further improvement. Despite the above strategies, small sample sizes may still lead to estimation bias, and the conclusions of this study need to be validated in external independent large-sample cohorts.

## 5. Conclusions

In summary, An exploratory correlation analysis revealed that both urea nitrogen and fibrinogen are factors associated with the prediction of GIB-HS in ICU patients, while APACHEII score, respiratory failure, and fibrinogen are independent factors affecting the prognosis of ICU patients with gastrointestinal bleeding and hemorrhagic shock. Clinicians need to identify these indicators in a timely manner and optimize treatment plans to improve prognosis.

## Supporting information

S1 Data The raw data for this study. (XLS)
